# Creutzfeldt-Jakob disease with unusually extensive neuropathology in a child treated with native human growth hormone 

**DOI:** 10.5414/NP300441

**Published:** 2012-02-20

**Authors:** Jacqueline Mikol, Jean-Philippe Deslys, Wen-Quan Zou, Wiangzhu Xiao, Paul Brown, Herbert Budka, Françoise Goutieres

**Affiliations:** 1 Denis Diderot University,; 2 Department of Pathology, Hopital Lariboisière, Paris,; 3 CEA/Institute of Emerging Diseases and Innovative Therapies, Fontenay-aux-­Roses, France,; 4 Department of Pathology, Case Western Reserve University, Cleveland, OH, USA,; 5 Institute of Neurology, Medical University of Vienna, and Austrian Reference Center for Human Prion Diseases, Vienna, Austria,; 6 No institutional affiliation

**Keywords:** growth hormone treatment, iatrogenic CJD, transitional form of panenecephalopathic CJD

## Abstract

We report a case of iatrogenic Creutzfeldt-Jakob disease(iCJD) in a child with a neonatal growth hormone (GH) deficiency that was treated with native human growth hormone (hGH) between the ages of 9 months and 7 years. Three years after the end of treatment a progressive neurological syndrome consistent with Creutzfeldt-Jakob disease (CJD) developed, leading to death within a year, at age 11. Neuropathological examination showed an unusual widespread form of CJD, notably characterized by (i) involvement of the cerebellar white matter, (ii) cortico-spinal degeneration and (iii) ballooned neurons. A transitional form of the disease between common iatrogenic and panencephalopathic CJD is suggested.

## Introduction**

In their review on “iatrogenic Creutzfeldt-Jakob disease (iCJD) at the millennium”, Brown et al. [1] emphasized some peculiarities according to the country of origin and showed that more than half of all growth hormone cases have occurred in France. 115 cases have been listed by 2010 [2]. Misfolded prion protein has been found in high concentration in the cerebellum of human growth-hormone (hGH) related iatrogenic CJD (iCJD), as opposed to some cases of sporadic CJD (sCJD), suggesting the existence of selection-specific strains in iCJD [3]. A recent biochemical study (2009) including the case reported here, has shown that, in contrast to sCJD, cases of iCJD have no detectable protease-resistant C-terminal fragment 12/13 [4]. This observation prompted us to re-investigate, histologically, our case, which had been briefly reported in an abstract in 1994 [5]. We found several histopathological hallmarks of the panencephalopathic form of CJD (PE-CJD) described by Mizutani [6, 7] principally seen in Japanese dura mater-associated iCJD [8] but in our case the hemispheric white matter lesions were less prominent. 

## Case report**

### Clinical summary

In January 1981, during the first week of life, a child of Algerian origin, presented a *status epilepticus* related to hypoglycemia secondary to panhypopituitarism with growth hormone deficiency. Due to persistent hypoglycemia, he had been treated between the age of 9 months and 6 years and 2 months (74 months) with cadaveric-derived human growth hormone, one injection every 3 days, and antiepileptic drugs, followed by synthetic hormone between the age of 7 and 10. Subsequently, at elementary school, poor psychomotor development with IQ assessed to be 40 was noted and cortical atrophy was present on the brain computerized tomography scan. Later on, the child was admitted to a specialized institution where he was considered as active and able to pronounce a few sentences. Nine years and 3 months after the first injection of native hormone, at the age of 10, he developed cerebellar ataxia and progressively became mute, confused and lost all his acquired knowledge. Neurological examination showed head and upper limb myoclonic jerks, pyramidal syndrome, unsteadiness and cerebellar ataxia, rigidity, voluntary vertical eye movements paresis with gaze dissociation. EEG recorded periodic sharp-wave complexes. Psychological studies confirmed the deterioration. Since October 1991, he was in a vegetative state and he died 13 months after the onset of symptoms at the age of 11 years and 3 months [9]. Autopsy was limited to the brain and the first segment of the cervical spinal cord excluding the pituitary gland. 

### Neuropathologic examination

**Figure 1:**
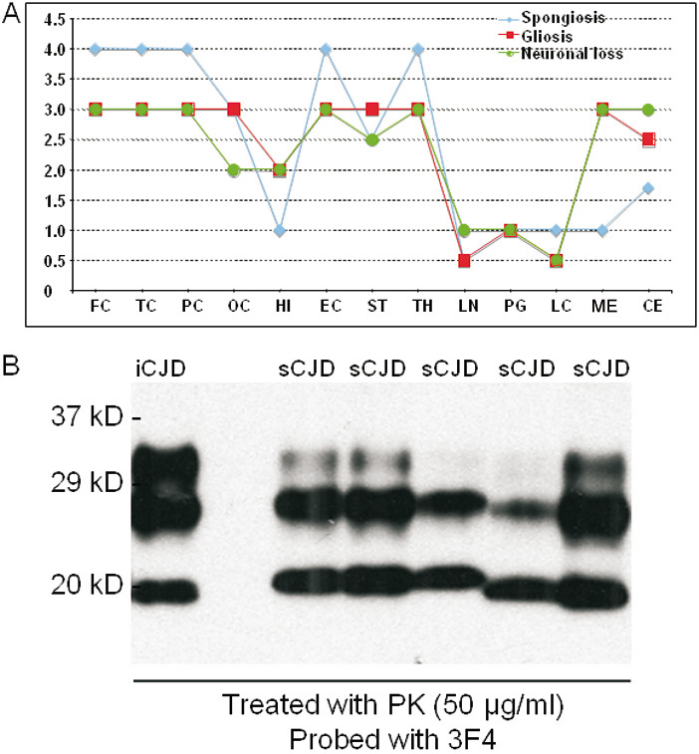
A: Gray matter lesion profiles: frontal (FC), temporal (TE), parietal (PC) and occipital (OC) neocortices, hippocampus (HI), entorhinal cortex (EC), striatum (caudatus and putamen nuclei) (ST), thalamus (TH), locus niger (LN), midbrain periventricular gray (PG), locus coeruleus (LC), medulla (ME), cerebellum (CB). B: Western blot analysis of PrP. Three sCJD with PrP Type 1 (MM1: 3 lanes at left) and two sCJD with PrP Type 2 (VV2: lanes at right) were used as controls.

Macroscopically the frontal and temporal lobes and the cerebellum were atrophic while the frontal ventricles were dilated. Histological examination was performed on sections of formalin fixed tissue, embedded in paraffin. The classical stains (hemalum-eosin, alcian blue, Nissl, Luxol-Fast-Blue, Periodic acid Schiff, Congo Red, Thioflavin S, Heidenhain, Kanzler and Bodian’s methods) were used as well as immunohistochemical techniques (antibodies against GFAP, ubiquitin, PrP, synaptophysin amyloid protein b, SMI 31 and 32, HSP 70, ab-crystallin SPA-223, Apo E, MBP and PLP). The degree of histopathological changes was scored from 0 to 3 or 4 (for spongiosis) according to Parchi [10] and the curves of neuronal loss, gliosis and spongiosis were parallel (Figure 1A). 

**Figure 2:**
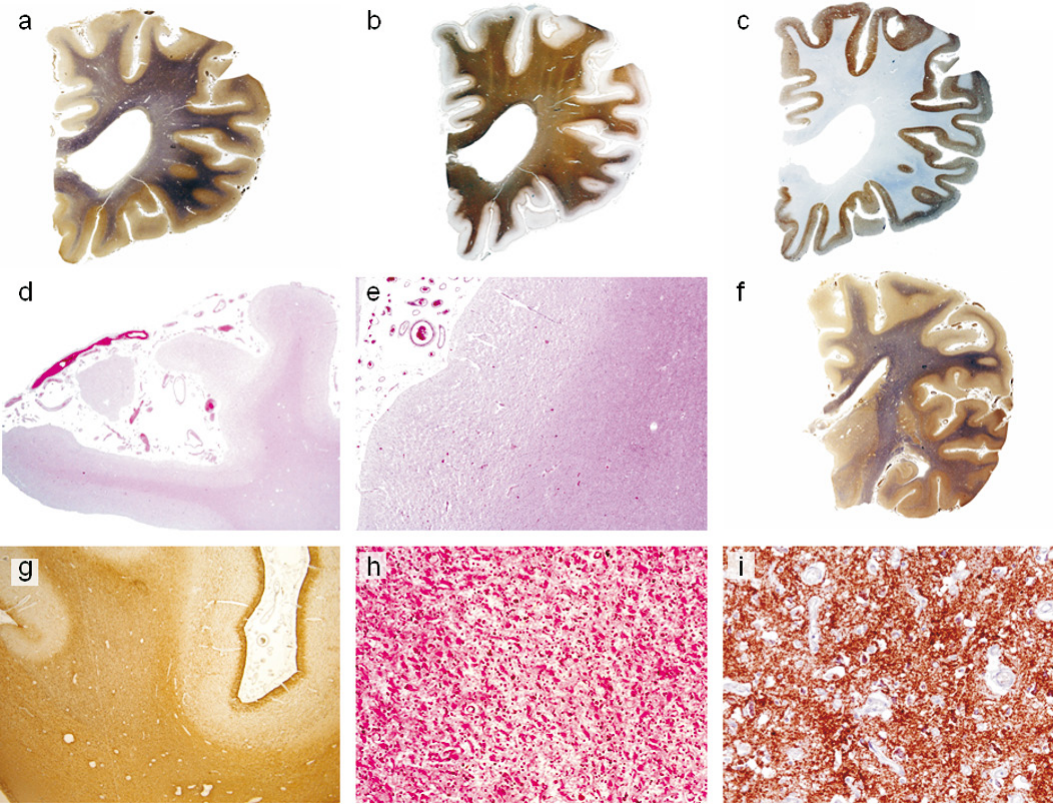
a, b, c: View of the left rostral frontal lobe showing cortical atrophy, dilatated ventricle and mild pallor of the white matter in Heidenhain stain (Hd). a: but less in immunostain for MBP (b) prominent PrP deposition in the frontal cortex (c). d: Atrophy of frontal gyri, hematoxylin-eosin (HE) stain × 2. e: Status spongiosus of the frontal cortex, HE stain × 25. f: Low- power view of hemispheric section showing atrophy of cortical ribbon and mild pallor of the white matter in Hd stain. g: Gliosis of frontal WM: immunostain for GFAP × 1.25. h: Massive neuronal loss and gliosis of putamen, HE stain × 62.5. i: Synaptic type PrP deposits in frontal cortex: immunostain with 3F4 antibody × 20.

**Figure 3:**
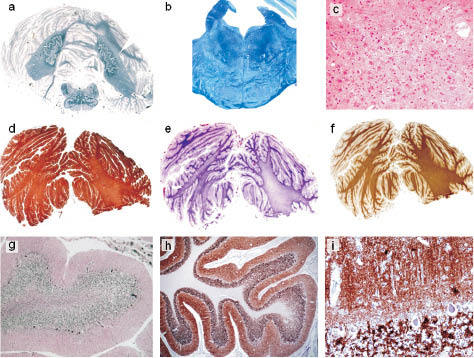
a: Massive atrophy of the cerebellum with white matter myelin loss. Degeneration of the cortico-spinal tracts, Hd stain × 0.5. b: Pons: degeneration of the cortico-spinal tracts, Luxol fast blue × 2. c: Detail of pons showing neuronal loss and astrocytosis of pontine nuclei: HE stain × 92. d: Gliosis of the lamellae and the album of the cerebellum: immunostain for GFAP. e: Comparison of the Kantzler stain for fibrillary gliosis and f: immunostain for MBP. g: Atrophic cerebellar folia, massive neuronal loss of granule cells, less pronounced of the Purkinje cells, and spongiosis, Bodian impregnation × 12. PrP deposits in the cerebellar cortex of synaptic type with a few plaque-like structures in h: × 2 and i: × 20.

Cortical atrophy (Figure 2a, b. c, d, f) and neuronal loss were massive in frontal, cingular, temporal, insular and claustral cortices, a little less noticeable in parietal and occipital cortices. The hippocampus was preserved, except for mild spongiosis in the molecular layer. Gliosis consisting of hypertrophic astrocytes paralleled neuronal loss, sometimes with a laminar distribution, and spongiosis conferred to the tissue a status spongiosus aspect (Figure 2e). Vacuolation involved all neocortical layers and was more pronounced in the superficial and the deep layers with laminar distribution; in the occipital gyrus the banding was also in the IV^th^ layer. In the underlying white matter, there was a very mild spongiosis but a diffuse, severe gliosis (Figure 2g) composed by gemistocytic astrocytes, with periventricular myelin loss and progressive radial fading, more prominent in Heindenhain’s staining but less in MBP immunostain (Figure 2a, b) and axonal depletion. There were no necrotic foci but minute patches of tissue rarefaction. The internal capsule was preserved. The typical histological triad of spongiform change, gliosis and neuronal loss was also observed in the basal ganglia, in particular the putamen (Figure 2h) and the thalamus. The putamino-pallidal tracts showed myelin loss. The lesions were also observed in the brain stem and cerebellum (Figure 3a) with massive neuronal loss, gliosis and moderate spongiosis (Figure 3c), especially in the pons with fiber loss of the ponto-cerebellar tracts (Figure 3b). The pyramidal tracts had fiber loss from the level of midbrain extending to the spinal cord where the involvement of the spinocerebellar tracts was more severe than that of the cortico-spinal tract. Neuronal loss was moderate in the pigmented nuclei. The cerebellum was also massively involved: the Purkinje cells and the granule cells had nearly disappeared (Figure 3g) as well as the neurons of the dentatus, emboliform and globosus nuclei while the spongiosis was mild to moderate. Pallor in the cerebellar white matter (Figure 3a) also indicated fiber loss while gliosis was more marked subcortically (Figure 3d, e). No amyloid deposits were demonstrated. 

**Figure 4:**
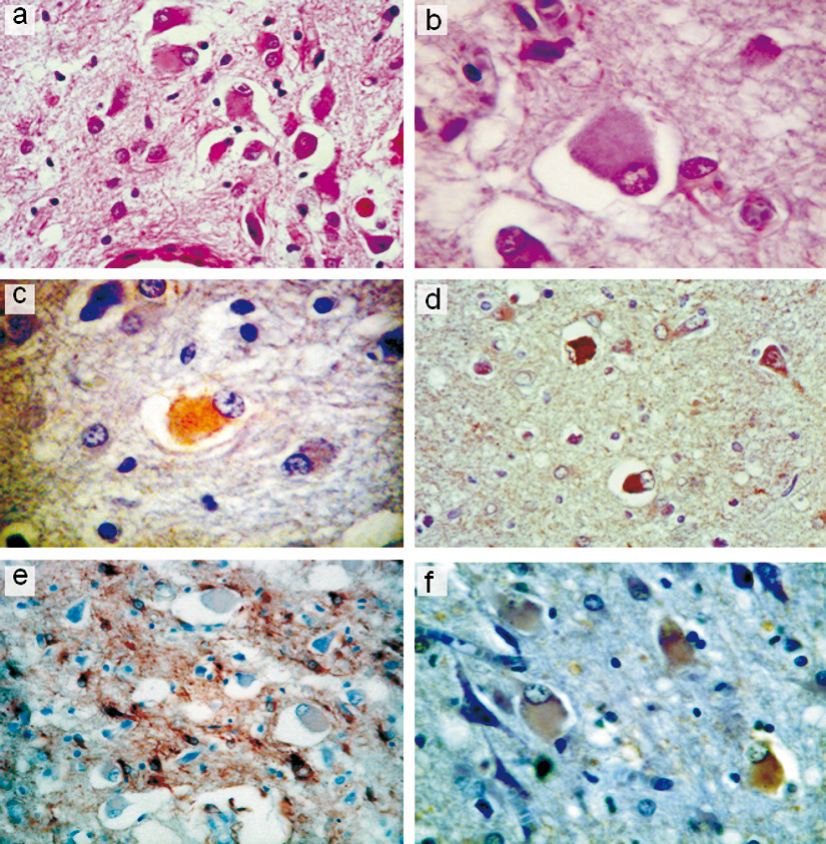
Ballooned neurons with excentric nucleus. a, b, c: cingular cortex, HE stain (a and b) and moderate positivity of the synaptophysin immunostain (c). d: Insular cortex: strong positivity with anti-phosphorylated neurofilament SMI 31. e: Cingular cortex: negative ballooned neurons are surrounded by astrocytes, immunostain for GFAP. f: Weak positivity of BN: immunostain for ubiquitin. a, d and e × 62.5; b, c, d × 125.

In addition, ballooned neurons (BN) were noted in the deep cortical layers; they were numerous in the cingular and frontal cortices, moderate in the lateral temporal lobe and rare in the insula, the parahippocampal gyrus and the parieto-occipital cortex. None was detected in the subcortical grey matter, the brain stem and the cerebellum. The BN had a pale eosinophilic swollen cytoplasm with no discernible Nissl substance and an eccentrically placed nucleus (Figure 4). A few exhibited slight argyrophilia but none had inclusions. The BN were mixed with hypertrophied astrocytes. 

Prion protein immunostaining revealed prominent (Figure 2c) diffuse and marked fine granular PrP deposit of the so-called synaptic type throughout the neocortex (Figure 2i). The labeling was slightly accentuated in the lower cortical layers with some prominent perineuronal staining. Some plaque-like PrP deposits were observed in the subcortical white matter. Strong positivity was also detected in the cerebellum including the cerebellar nuclei. Moreover, in the granular layer of the cerebellum patchy small plaque-like PrP deposits (Figure 3i) were also positive for Apo E. No deposit was congophilic. The BN were positive for SMI 31, synaptophysin, ubiquitin and HSP 70, and negative for ab-crystallin and GFAP (Figure 4). Expression of excitatory amino-acid transporter 1 (EAAT1) in brain macrophages and microglia, studied previously, was mild [11]. 

### PrP gene analysis and western blot analysis of prion protein

Genomic DNA was extracted from frozen tissue and used to amplify the open reading frame of the PrP gene by PCR. RFLP analysis and sequencing revealed valine homozygosity at codon 129 and no pathogenic mutations. 

Brain tissue from the frozen frontal lobe was homogenized and western blot analysis was performed with anti-3F4 antibody as described previously [12, 13]. The result was positive for the presence of proteinase K-resistant PrP with gel mobility of the unglycosylated PrP band migrating at 19 kDa (Figure 1B), the molecular signature of PrP Type 2 in sCJD [14]. Although the gel mobility of PrP was similar to that of sCJD, the glycoform ratios were different. Compared to sCJD Type 2 with Met/Met polymorphism at residue 129, the ratio of the diglycosylated to monoglycosylated PrP was increased (Figure 1B). However, it was similar to that of sCJD Type 2 with 129 Val/Val [10]. Moreover, the PK-resistant PrP terminal fragments (PrP-CTF 12/13) [4] were not detectable (data not shown), whereas they were detected in sCJD [15]. 

## Discussion 

When previously investigated, our patient, one of the first French hGH cases, was considered to have a characteristic form of iCJD appearing after an incubation period between 3 and 8.5 years: the clinical features were consistent with the descriptions of other French cases [16], and the patient was homozygous for valine at the polymorphic codon 129, which is over-represented in iCJD due to hGH [17, 18]. He was one of the patients treated at the earliest age, and his contamination probably took place in the critical period of contaminated French hGH, between 1984 and 1985 [19] when he was 3 – 4 years old. Our patient survived for 1 year, i.e. a long clinical course in a very young boy. Age had previously been noted as determinant in the duration of sporadic CJD [20, 21]. 

The pattern of neuropathological changes supported the diagnosis of iCJD [22, 23] but was really unusual. The cortex was severely affected, and massive gliosis was observed in the white matter (WM) which involved the complete brain (mild in periventricular cerebral WM and more prominent in cerebellar WM, documented by diffuse pallor on myelin stains, as well as degeneration of the cortico-spinal tracts at the level of the brainstem and the cervical spinal cord). The WM involvement was reported as a characteristic feature of panencephalopathic CJD (PE-CJD): more than 70 cases of PE-CJD have been reported so far including, at least, 48 Japanese cases. Many of them were sporadic, few were familial, and, in Japan, most of them secondary to dura mater grafts [8]. Degeneration of the pyramidal tract was mostly investigated in the Japanese forms (essentially MM1 patients) [6, 7, 24]: it was observed from the cerebral WM to the lumbar spinal cord or was limited to the lumbosacral segment. In our patient, the degeneration was seen in the brainstem and the cervical spinal cord (the only level examined). According to Iwasaki et al. [24] the degeneration would be the result of a “distal-dominant” pathology. 

The origin of WM involvement in CJD is arguable. Some authors consider it to be a secondary consequence of neuronal loss [25] (for review see [26]); others consider the intense or even massive aspect of the lesions [27] and the large number of gemistocytic astrocytes, to favor a primary character of the WM involvement [28, 29]. Paradoxically, after corticoid treatment of a clinical case, diagnosed by Flair neuroimaging, there was complete and unusual resolution on the WM changes on images [30], suggesting resolution of an inflammatory or oedematous component. Whatever might be the pathogenesis,the peculiar neuropathological pattern of the disease in our patient suggests a transitional form between (polioencephalopathic) iCJD and PE-CJD. Interestingly, WM vacuolation related to neuronal loss has also recently been reported in vCJD [31]. 

The ballooned neurons present in our case were labeled by SMI 31, a marker of phosphorylated neurofilaments, suggesting a common mechanism of impaired axoplasmic transport associated with activation of the ubiquitin proteome system [32]. BN are seen in several neurodegenerative diseases of the central nervous system [32, 33]. However, BN were different from the swollen neurons of pellagra with central chromatolysis. The relationship with focal dysplasia of Type II with BN was also ruled out as no dysplasia was demonstrated and neuronal markers were positive for BN [34, 35]. Indeed BN are part of prion disease pathology. Similar swollen neurons have already been illustrated in the cases of Creutzfeldt and Jakob and described in patients suffering from CJD [10]; they are especially frequent in Japanese patients with a strong predominance in PE-CJD [6, 7, 36]. In contrast, BN seem to be rare in non-Japanese cases of PE-CJD [37] but in a case of prion dementia without characteristic pathology, related to an insertional *PRNP* aberration, a few BN were already seen in the cortex [38]. 

Concerning the genetic background, the relation between the polymorphism at *PRNP* codon 129 and PrP ^res^ isotypes has been studied in a few recent cases. According to Parchi et al. [10], most of them belonged to the MM1 or MM2 subgroups and a few were MV [24, 25, 26]. Our case was VV at codon 129 with no mutation like many of the French iatrogenic cases [18]. It was included as biochemical Type 2 in the series subjected to molecular biological analysis by Yuan et al. [4] who proposed a method to separate iCJD from sCJD. Very limited neuropathological data characteristic of VV2 subtype from Parchi et al. [14] was observed in our case: neocortex was massively involved and spongiosis was not limited to deep layers; PrP staining showed limited plaque-like deposits and more perineuronal staining. These lesions combine those observed in iCJD [23] but include other areas involved in PE-CJD associated with Type 1 which suggest a transitional form between these profiles. 

This combination of neuropathological features in our case seems to be peculiar. They do not indeed fulfil all criteria of the rare PE-CJD [6, 7] because the myelin involvement is moderate but degeneration of pyramidal tract and ballooned cells were present. In consequence, we propose here a transitional form of the disease, between common iCJD and PE-CJD. The young age of the patient and the preexisting panhypopituitarism may have contributed to the unusually long duration of the disease and to the peculiar neuropathology observed. To define the mechanisms of WM involvement, further studies will still be needed. The possible selective evolution of a specific prion strain in this unusual variety of hGH-associated iCJD might also have to be considered. 

## Acknowledgments 

The technical expertise of C. Poiron, P. Castagnet, E. Dirnberger, I. Leisser, J. Dellanave and S. Freire is gratefully acknowledged. Work supported in part by grants (to W.Q.Z.) from the CJD Foundation, the NIH RO1NS062787, the foundation Alliance Biosecure and the University Center on Aging and Health, with the support of the McGregor Foundation and the President’s discretionary Fund (Case Western Reserve University).
